# Comparative Genomic Analysis of the Lettuce Bacterial Leaf Spot Pathogen, *Xanthomonas hortorum* pv. *vitians*, to Investigate Race Specificity

**DOI:** 10.3389/fmicb.2022.840311

**Published:** 2022-04-18

**Authors:** Emma Rosenthal, Neha Potnis, Carolee T. Bull

**Affiliations:** ^1^Department of Plant Pathology and Environmental Microbiology, Pennsylvania State University, University Park, PA, United States; ^2^Department of Entomology and Plant Pathology, Auburn University, Auburn, AL, United States

**Keywords:** bacterial plant pathogens, plant-microbe interactions, comparative genomics, *Xanthomonas*, effectors

## Abstract

Bacterial leaf spot (BLS) of lettuce caused by *Xanthomonas hortorum* pv. *vitians* (*Xhv*) was first described over 100 years ago and remains a significant threat to lettuce cultivation today. This study investigated the genetic relatedness of the *Xhv* strains and the possible genetic sources of this race-specific pathogenicity. Whole genome sequences of eighteen *Xhv* strains representing the three races, along with eight related *Xanthomonas* strains, were included in the analysis. A maximum likelihood phylogeny based on concatenated whole genome SNPs confirmed previous results describing two major lineages of *Xhv* strains. Gene clusters encoding secretion systems, secondary metabolites, and bacteriocins were assessed to identify putative virulence factors that distinguish the *Xhv* races. Genome sequences were mined for effector genes, which have been shown to be involved in race specificity in other systems. Two effectors identified in this study, *xopAQ* and the novel variant *xopAF2*, were revealed as possible mediators of a gene-for-gene interaction between *Xhv* race 1 and 3 strains and wild lettuce *Lactuca serriola* ARM-09-161-10-1. Transposase sequence identified downstream of *xopAF2* and prophage sequence found nearby within *Xhv* race 1 and 3 insertion sequences suggest that this gene may have been acquired through phage-mediated gene transfer. No other factors were identified from these analyses that distinguish the *Xhv* races.

## Introduction

Bacterial leaf spot (BLS) of lettuce is caused by *Xanthomonas hortorum* pv. *vitians* (*Xhv*) Morinière et al. (2020) (formally *X. campestris* pv. *vitians*), which was first described in 1918 following an outbreak in South Carolina lettuce fields (Brown, 1918). BLS is a sporadic, yet significant threat to lettuce production worldwide, and several subsequent outbreaks in the last century have prompted research to better understand this plant-pathogen interaction. Investigation into *Xhv* strain diversity has been vital to the development and application of effective strategies for the management of BLS. In 2015, Bull et al. (2015) demonstrated that some, but not all, of the 120 *Xhv* strains evaluated resulted in a hypersensitive response (HR) to limit the spread of infection upon inoculation into *Lactuca sativa* cvs. Little Gem, Pavane, and La Brillante, and that these strains did not cause HR in the other lettuce cultivars that were tested. This subset of *Xhv* strains was designated race 1 to correspond to the HR elicited in Little Gem, Pavane, and La Brillante and the hypothesized interaction with the resistance gene (R-gene) *Xar1* mapped to chromosome 2 in these cultivars (Hayes et al., 2014). Another identified source of resistance to *Xhv* race 1 strains isolated from Florida lettuce fields was the *Xcvr* R-gene from *L. serriola* PI358001-1 (Wang et al., 2016). The presence of these R-genes led to the hypothesis that *Xhv* race 1 strains engage in a gene-for-gene interaction with these lettuce cultivars. Additional races of the pathogen were designated among those *Xhv* strains that did not result in HR in the *Xar1-* or *Xcvr*-encoding cultivars, but instead triggered HR in either *L. serriola* PI491114 (designated race 2) or *L. serriola* ARM-09-161-10-1 (designated race 3). Recent HR screening also suggests that *Xhv* race 1 strains might also induce HR in ARM-09-161-10-1 (Rosenthal et al., unpublished).

Bull et al. (2016) found that the diversity they observed in the resistance phenotypes of the lettuce cultivars corresponded to the genetic diversity of the pathogen. They demonstrated the presence of five distinct sequetypes among 120 *Xhv* strains using a multi-locus sequence analysis (MLSA) scheme originally designed for distinguishing *Xanthomonas* spp. (Maiden et al., 1998; Young et al., 2008). The results of this study corroborated previous work on *Xhv* diversity (Sahin et al., 2003; Fayette et al., 2016), and they found that the strains that induce HR upon injection into the *Xar1-* or *Xcvr*-encoding cultivars all belonged to sequetypes B, D, or E. The strains which triggered HR in PI491114 all belonged to sequetype A, and those that triggered HR in ARM-09-161-10-1 all belonged to sequetype C. These results supported the hypothesis that genotypic differences underly the differences in resistance phenotype. The relationship between sequetype and race also supported the finding that *Xhv* populations are predominantly clonal, though with some sequence variation due to recombination (Fayette et al., 2016).

Studies of plant-pathogen interactions have suggested a constant cycle in which plants and their pathogens evolve to outperform each other, in defense strategy and pathogenicity, respectively (Dangl and Jones, 2001; Zipfel and Felix, 2005). Pathogen presence can trigger basal plant defenses to prevent further colonization, which can be then interrupted by the delivery of bacterial effector proteins through the type three-secretion system. In turn, effector activity can be thwarted by plant host recognition of those effectors and activation of resistance (R-)genes, such as those that activate HR to kill off invaded tissues and limit the spread of infection. The presence of the R-genes *Xar1* and *Xcvr* in lettuce cultivars capable of HR exclusively to *Xhv* race 1 (Hayes et al., 2014; Wang et al., 2016) suggested the possibility that *Xhv* race 1 might produce an effector that is recognized by those R-genes. Other gene-for-gene interactions might also be at play for *Xhv* races 2 and 3 and their respective HR-inducible lettuce cultivars, though R-genes have not yet been demonstrated for PI491114 and ARM-09-161-10-1.

In other bacterial plant pathogens, the proximity of many type III effectors (T3Es) to mobile genetic elements suggests that they were acquired or lost through horizontal gene transfer with other bacteria in their local microbiome (McCann and Guttman, 2008; Merda et al., 2017). Following effector gene acquisition or loss, the selective environment created by the host’s defenses may have caused the new genotype to be maintained and to be host-specific (Sarkar et al., 2006; Hajri et al., 2009) or even race-specific. Such a relationship between race-specific genotype and a tomato cultivar has already been demonstrated for *X. euvesicatoria* pv. *perforans* (Astua-Monge et al., 2000).

Other gene products, such as secondary metabolites and bacteriocins, have been shown to play a role in virulence. The *Xanthomonas* pigment Xanthomonadin and siderophore Xanthoferrin contribute to bacterial fitness by protecting bacterial cells from UV damage and sequestering iron in low-iron conditions, respectively; functional knockouts of these gene clusters resulted in reduced virulence according to Rajagopal et al. (1997) and Pandey et al. (2017). A variety of bacteriocins identified in *Xanthomonas* spp. enhance bacterial competitiveness through antimicrobial activity (Holtsmark et al., 2008). Although a role in host-specific pathogenicity for these components has not yet been demonstrated, it is possible that race-specific inclusion of these genes may be used to further distinguish the *Xhv* races.

This study was designed to investigate variation in effector repertoires between the *Xhv* races as a possible genetic source for race-specific HR elicitation. Several important virulence factors were also studied to look for genotypic differences that could be used to distinguish the *Xhv* races. Eighteen geographically diverse isolates of *Xhv* and seven related plant pathogenic *Xanthomonas* strains were sequenced and assembled. A genome assembly for *X. hortorum* pv. *carotae* M081 was also retrieved and included for comparison. Genome statistics and predicted features, including plasmids and secretions systems, were determined for each strain. A maximum likelihood phylogeny based on SNP data corroborated the taxonomic relationships predicted by MLSA (Bull et al., 2016; Fayette et al., 2016). Mining the genomes for effector sequences and other potential virulence factors revealed insights into the race-specificity of the BLS pathogen. A preliminary report of this data has been given (Rosenthal et al., 2018).

## Methods

### Bacterial Strains and Culturing Methods

All strains used in this study and their sources are shown in Table 1. Eighteen strains of *Xhv* were selected as representatives of the three pathogenic races designated in previous studies: race 1 (12 strains), race 2 (4 strains), and race 3 (2 strains). The type and pathotype strains of *X. hortorum* pv. *hederae, X. hortorum* pv. *taraxaci, X. hortorum* pv. *pelargonii, X. hortorum* pv. *gardneri*, *and X. hortorum* pv. *cynarae* were included for comparison to the *Xhv* strains, as well as one *Xanthomonas hortorum* strain from radicchio. *X. campestris* pv. *coriandri* was included as an outgroup. Bacteria were routinely cultured on nutrient agar (NA) and in nutrient broth (NB), both at room temperature (20–28^°^C).

**TABLE 1:** *Xanthomonas* strains included in this study.

Organism	Strain designations	Other strain IDs	Geographic origin	Host of isolation (common name)	Race*	Source or citation
*X. hortorum* pv. *vitians*	BP5172	*Xav* 98-37 2/01	Salinas, CA, United States	*L. sativa* (lettuce)	1	J. Barak
	BS0339	Salinas 2/01	Salinas, CA, United States	*L. sativa* (lettuce)	1	J. Barak
	BS0340	*Xav* 98-23 2/01	Salinas, CA, United States	*L. sativa* (lettuce)	1	J. Barak
	BS0347	*Xcv* 5/01	Salinas, CA, United States	*L. sativa* (lettuce)	1	J. Barak
	BP5176	*Xcv* 5/01	Salinas, CA, United States	*L. sativa* (lettuce)	1	J. Barak
	BP5177	“Edge A”	Colorado, United States	*L. sativa* (lettuce)	1	S. Koike
	BP5179	“Daniel Rom”	Salinas, CA, United States	*L. sativa* (lettuce)	1	S. Koike
	BP5182	“Moreno Let”	Santa Maria, CA, United States	*L. sativa* (lettuce)	1	S. Koike
	NCPPB 4058	N/A	United Kingdom	*L. sativa* (lettuce)	1	H. Stanford
	CFBP 8686^PT^	LMG 938^PT^, NCPPB 2248^PT^, MR20213^PT^	Zimbabwe	*L. sativa* (lettuce)	1	Vauterin et al., 1995; Morinière et al., 2020
	BP5191	VT111	Canada	*L. sativa* (lettuce)	1	V. Toussaint
	BP5192	*Xcv*-2	CA, United States	*L. sativa* (lettuce)	1	C. T. Bull
	ICMP 1408	PDDCC 1408	Ithaca, NY, United States	*L. sativa* (lettuce)	2	W. H. Burkholder
	ICMP 4165	LMG 7508, PDDCC 4165	New Zealand	*L. sativa* (lettuce)	2	H. J. Boesewinkel
	BS3127	VT106	Canada	*L. sativa* (lettuce)	2	V. Toussaint
	BP5194	917	Ohio	*L. sativa* (lettuce)	2	Sahin et al., 2003
	BS2861	“Christy BuLet 2”	King City, CA, United States	*L. sativa* (lettuce)	3	S. Koike, Rianda
	BP5181	“Christy BuLet 3”	King City, CA, United States	*L. sativa* (lettuce)	3	S. Koike, Rianda
*X. hortorum* from radicchio	BP5178	N/A	Salinas, CA, United States	*Cichorium intybus* (radicchio)		Zacaroni et al., 2012
*X. hortorum* pv. *hederae*	CFBP 4925^T^	ICMP 453^T^, NCPPB 939^T^, LMG 733^T^	United States	*Hedera helix* (English ivy)		Arnaud, 1920; Dye, 1978; Vauterin et al., 1995
*X. hortorum* pv. *taraxaci*	CFBP 410^PT^	ATCC 19318^PT^, NCPPB 940^PT^, LMG 870^PT^	Ithaca, NY, United States	*Taraxacum kok-saghyz* (Russian dandelion)		Niederhauser, 1943; Dye, 1978; Vauterin et al., 1995
*X. hortorum* pv. *pelargonii*	CFBP 2533^PT^	ICMP 4321^PT^, LMG 7314^PT^, NCPPB 2985^PT^	Auckland, New Zealand	*Pelargonium peltatum L. L’Hér.* (pelargonium)		Brown, 1923; Dye, 1978
*X. hortorum* pv. *gardneri*	CFBP 8163^PT^	LMG 962^PT^, ATCC19865^PT^, NCPPB 881^PT^, PDCC 1620^PT^	Yugoslavia	*Lycopersicon esculentum* Mill. (tomato)		(ex Šutić 1957) Jones and Dangl, 2006
*X. hortorum* pv. *cynarae*	CFBP 4188^PT^	ICMP 16775^PT^	France	*Cynara scolymus* (artichoke)		Trébaol et al., 2000.
*X. hortorum* pv. *carotae*	CFBP 7900	M081	Hungary	*Daucus carota* L. var. sativus (carrot)		Kendrick, 1934; Dye, 1978; Vauterin et al., 1995
*X. campestris* pv. *coriandri*	CFBP 8452^PT^	LMG 687^PT^, ATCC 17996^PT^, ICMP 5725^PT^, NCPPB 1758^PT^, PDDCC 5725^PT^	India	*Coriandrum sativum* (coriander)		Srinivasan et al., 1961; Dye, 1978; Vauterin et al., 1995

*Type and pathotype strains are marked T and PT, respectively, in the strain designation column.*

*There are currently 10 Xhv race 2 strains and four Xhv race 3 strains described.

### DNA Extraction and Whole Genome Sequencing

DNA extraction was performed using the Qiagen DNeasy UltraClean Microbial Kit (cat. no. 12224; Valencia, CA, United States) according to the manufacturer’s instructions, with the following change: five replicates were completed for each strain and after eluting each first replicate, that elute was used to elute the next replicate, and so on. This resulted in >2.0 μg of total DNA per strain, as measured using the Thermo Fischer Qubit Fluorometer 3.0 and the Invitrogen dsDNA Broad Range Assay Kit (cat nos. Q33216 and Q32850; Waltham, MA, United States). Library preparation and 250 × 250 paired-end whole genome sequencing was performed at the Pennsylvania State University Genomics Facility using the TruSeq DNA PCR Free Library Construction Kit and the Illumina MiSeq System (cat. nos. FC-121-3001 and SY-410-1003; San Diego, CA, United States).

### Quality Control and Whole Genome and Plasmid Assembly

Trimmomatic PE v.0.39 (Bolger et al., 2014) was used to remove Illumina adapters and poor-quality sequence, defined as four consecutive bases with phred scores lower than 20 and DNA fragments shorter than 50 bases. Sequence quality reports were generated using FastQC v0.11.9 (Andrews, 2010) before and after using Trimmomatic PE to verify an improvement in sequence quality which would improve the accuracy of the genome assembly. This procedure was completed in a loop using trimming.sh, spades.sh, and spades_loop.txt (Supplementary Data 1–3).

Trimmed reads were assembled *de novo* using SPAdes v3.12.0 (Bankevich et al., 2012) and Quast v5.0.0 (Gurevich et al., 2013) was used to provide descriptive statistics about the assemblies (Table 2). Plasmid counts were predicted using the Recycler pipeline (Rozov et al., 2017) and plasmid sequences were predicted using plasmidSPAdes (Antipov et al., 2019); using both programs for plasmid analysis has been recommended by the creators of plasmidSPAdes due to the differing predictive strengths of each tool. Raw reads and whole genome sequence assemblies are available at BioProject PRJNA790934. Completeness of the whole genome assemblies was assessed using BUSCO v5.2.2 (lineage = xanthomonadales_odb10 and mode = genome; Manni et al., 2021) to compare these assemblies against several publicly available genome assemblies for related *Xanthomonas* strains obtained from NCBI’s genome database, including *X. campestris* pv. *campestris* (ATCC 33913^T^), *X. populi* (CFBP 1817^T^), *X. arboricola* pv. *juglandis* (CFBP 2528^T^), *X. hortorum* pv. *pelargonii* (CFBP 2533^PT^), *X. hortorum* pv. *taraxaci* (CFBP 410^PT^), *X. hortorum* pv. *cynarae* (CFBP 4188^PT^), *X. hortorum* pv. *carotae* (CFBP 7900), *X. hortorum* pv. *gardneri* (CFBP 8163^PT^), *X. hortorum* pv. *vitians* (CFBP 8686^PT^), *X. campestris* pv. *coriandri* (ICMP 5725^PT^), *X. fragariae* (PD 885^T^), and *X. hortorum* pv. *hederae* (WHRI 7744^T^).

**TABLE 2:** Genome characteristics for *Xanthomonas hortorum* pv. *vitians* strains and related type and pathotype strains.

	Strain	Genome size (Mb)	N50	GC%	Contig no.	CDSs no.	CDSs Length (Mb)	% Genome CDS	Plasmid no.	ISs compared to BS3127
*X. hortorum* pv. *vitians* race 1	BP5172	5.1	90636	63.7	128	4590	4.6	91.1	2	97
	BS0339	5.2	45509	63.6	223	4747	4.7	90.8	1	93
	BS0340	5.4	82765	63.4	324	4653	4.7	86.7	0	105
	BS0347	5.1	114952	63.7	114	4646	4.7	91.0	0	91
	BP5176	5.1	40310	63.7	264	4644	4.7	91.4	3	72
	BP5177	5.2	89202	63.7	138	4674	4.7	90.8	1	104
	BP5179	5.2	26996	63.7	364	4702	4.7	91.0	4	87
	BP5182	5.2	55827	63.7	179	4712	4.7	90.9	2	94
	NCPPB 4058	5.3	80295	63.6	130	4793	4.8	90.6	0	95
	CFBP 8686^PT^	5.1	53428	63.8	183	4591	4.6	91.4	2	88
	BP5191	5.3	49126	63.5	220	4848	4.8	90.8	1	64
	BP5192	5.1	106975	63.7	130	4665	4.7	91.0	0	100
*X. hortorum* pv. *vitians* race 2	ICMP 1408	5.2	107536	63.7	109	4720	4.7	91.3	0	0
	ICMP 4165	5.3	129151	63.6	107	4865	4.9	91.2	1	0
	BS3127	5.1	106407	63.7	98	4647	4.7	91.6	0	0
	BP5194	5.2	33155	63.6	271	4725	4.7	91.5	0	0
*X. hortorum* pv. *vitians* race 3	BS2861	5.2	118085	63.7	120	4690	4.7	90.9	0	no data
	BP5181	5.2	98381	63.7	128	4684	4.7	91.0	1	111
*X. hortorum* from radicchio	BP5178	5.2	58212	63.7	199	4655	4.7	91.1	1	no data
*X. hortorum* pv. *hederae*	CFBP 4925^T^	5.4	43552	63.8	270	4916	5.0	91.7	1	no data
*X. hortorum* pv. *taraxaci*	CFBP 410^PT^	5.0	53166	63.9	173	4535	4.6	92.5	1	no data
*X. hortorum* pv. *pelargonii*	CFBP 2533^PT^	5.2	128949	63.8	83	4788	4.9	92.9	1	no data
*X. hortorum* pv. *gardneri*	CFBP 8163^PT^	5.2	112448	63.7	95	4707	4.7	91.5	1	no data
*X. hortorum* pv. *cynarae*	CFBP 4188^PT^	5.4	129044	63.4	335	4633	4.7	87.3	1	no data
*X. campestris* pv. *coriandri*	CFBP 8452^PT^	5.1	422303	64.9	38	4294	4.4	87.1	0	no data

### Variant-Based Phylogeny Using Whole Genome Sequences

To investigate the genetic relatedness of the *Xhv* strains and related strains used in this study, a phylogenetic tree was constructed based on a core alignment of single nucleotide polymorphisms (SNPs). The whole genome assembly of *Xanthomonas hortorum* pv. *carotae* (*Xhc*) M081 was downloaded from NCBI (GenBank assembly accession GCA_000505565.1) to be used for comparison. The snippy-multi pipeline v4.3.6 (Seemann, 2015) was used to map trimmed Illumina sequencing reads to the *Xhc* M081 reference and to identify and align a set of core SNPs. In the resulting core alignment, all non-[AGTCN-] characters were replaced using snippy-clean_full_aln and then loci with high levels of base substitution, indicating possible recombination, were removed using run_gubbins.py v2.4.1 (Croucher et al., 2015). SNPs were extracted from the filtered multi-fasta alignment using SNP-sites with the option “-c” to only output AGTC into a final alignment file. This file was imported into CLC Genomics Workbench and General Time Reversible with rate variation (four categories) and estimated topology was selected as the appropriate substitution model following model testing with the neighbor-joining clustering method. This model was used to produce a maximum-likelihood phylogeny with 1,000 bootstraps and branch lengths that represent the expected number of nucleotide substitutions per sequence site.

### Effector Gene Mining

BLAST databases were constructed from each whole genome assembly scaffold file or plasmid scaffold file. A FASTA file containing known bacterial effector protein sequences, including those previously published for plant pathogenic *Xanthomonas* and *Pseudomonas* species, was provided (White et al., 2009). Translated nucleotide BLAST was used to align each entry of this effector sequence catalog to the whole genome sequence databases. Matches were filtered to include those with greater than 60% identity, greater than 40% query coverage per high scoring sequence pair, and less than 0.0001 expect value. Other potential matches were found using a filter of greater than 45% identity, greater than 40% qcovhsp, and less than 0.00001 e-value; this allowed for inclusion of some other effectors which may be present but with moderate sequence variation compared to those in our database. The same searches were performed using the predicted plasmid sequences to determine which effectors may be carried on plasmids. The filtered matches constitute the hypothesized effector repertoires of that strain. Mining for these effector repertoires was completed using the script effectorgene_mining.sh (Supplementary Data 4).

### Gene Alignments and Trees

Nucleotide sequences for two genes of interest, *xopAQ* and *xopAF*, were extracted from our assembly files using the program samtools v1.9 faidx and the gene positions found from the effector gene mining procedure (Li et al., 2009). These sequences were used as input for the ORFfinder program available online at https://www.ncbi.nlm.nih.gov/orffinder/, and the longest ORFs were selected for protein alignment using MEGA11: Molecular Evolutionary Genetics Analysis (Tamura et al., 2021), along with the original protein sequences published for the homologs XopAQ, HopAQ1, Rip6, and Rip11 (Guttman et al., 2002; Mukaihara et al., 2010; Potnis et al., 2011); or XopAF (AvrXv3) and HopAF1 (Astua-Monge et al., 2000; Petnicki-Ocwieja et al., 2002). Overhanging sequences were trimmed. Maximum-likelihood phylogenies were generated from these alignments to show the relatedness of the extracted genes. The Jones-Taylor-Thornton (JTT) amino acid substitution model, an assumption of uniform substitution rates among sites, and the nearest-neighbor-interchange (NNI) method were used to generate the phylogenies. The phylogenies were tested using 1,000 bootstraps and branch lengths represented the number of amino acid substitutions per site.

### Mobile Genetic Elements

Mobile genetic elements were assessed for their effector content, especially for *xopAQ* and *xopAF* presence, to find evidence of recent acquisition by a common ancestor of *Xhv* race 1 and 3 strains. Large insertion sequences in *Xhv* race 1 and 3 strains were identified by aligning their whole genome sequencing reads to the assembled sequence for *Xhv* race 2 strain BS3127, identifying the sequence regions present in their genomes that were not present in BS3127, and aligning the regions flanking the putative insertion to their whole genome assemblies to find the complete insertion sequences. These steps were completed following the MGEfinder pipeline (Durrant et al., 2020). *Xhv* race 3 strain BS2861 was excluded from this analysis because its inclusion caused the program to fail. The identified insertion sequences were then mined for effector genes using the method described above, as well as for transposase sequences included in the ISEScan package (Xie and Tang, 2017) using the same method and phage elements using PHASTER’s online platform (Arndt et al., 2016) with the default settings.

### *xopAF* and *xopAQ* Gene Regions in *Xhv* Race 2 Strains

Whether there were other known genes present at the sites in *Xhv* race 2 strains where *xopAF* and *xopAQ* were absent was analyzed. Sequences flanking the *xopAF* and *xopAQ* genes from BP5172 (*Xhv* race 1) were extracted using samtools faidx, including 1,000 nucleotides on either side. *Xhv* race 2 strains were then parsed for these sequences using BLAST to identify the same regions of their genome sequence. Alignments of these regions were completed for a subset of *Xhv* race 1 and 2 strains, and both the *Xhv* race 3 strains. These alignments revealed the sequence within the *Xhv* race 2 genomes that were present instead of the *xopAF* and *xopAQ* genes. These sequences were copied into NCBI’s ORFfinder to look for coding sequence and NCBI’s Conserved Domain Search to determine the possible function of that coding sequence. One protein-coding gene of interest which was present in all *Xhv* race 2 strains instead of *xopAF* was used as a query in a BLAST search of *Xhv* race 1 and 3 genomes to determine whether this gene was specific to *Xhv* race 2 strains or present in all *Xhv* strains tested.

### Secretion System Gene Mining

Genes associated with or encoding secretion system components were compiled into a database by downloading their sequences from representative species from NCBI (Pena et al., in preparation). Nucleotide BLAST using the script SSgene_mining.sh (Supplementary Data 5) revealed genes from our database which aligned to the whole genome sequence assemblies. Plasmid sequences were also queried for secretion system genes. Matches were filtered to retain only those that had 65% identity and 65% query coverage per high scoring sequence pair or higher and an e-value less than 0.00001. To confirm these results, additional global alignments of extracted secretion system gene clusters were completed in CLC Genomics Workbench, and aligned genes were again filtered for 65% identity. Reported results represent the sum of these two methods.

### Secondary Metabolite/Bacteriocin Gene Mining

Secondary metabolite gene clusters were predicted for each strain using the antiSMASH v5.0 online server using the “relaxed strictness” parameter and the “KnownClusterBlast” feature (Blin et al., 2019). Individual genes within these clusters were manually filtered to include only hits with greater than 60% identity and 40% query coverage.

To verify the antiSMASH results, each whole genome assembly was also uploaded to the BAGEL4 webserver and queried against a database of known bacteriocin genes and motifs using default settings (van Heel et al., 2018). Areas of interest that were scored as significant by the program (where the sum of weight factors is greater than 175) are reported here along with their identified class.

## Results and Discussion

### Comparative Genomic Analysis

**FIGURE 1:**
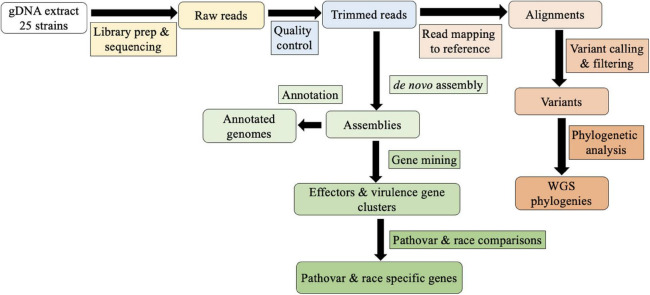
Bioinformatics workflow for comparing whole genome sequences.

Eighteen assembled genomes representing the three pathogenic races of *Xhv*, as well as those of seven related *Xanthomonas* strains, are available at BioProject PRJNA790934. The bioinformatics pipeline used in this study is shown in Figure 1. Genome-associated statistics are reported in Table 2. The average *Xhv* genome size was 5.2 Mbp and in this study the number of contigs ranged from 98 for the *Xhv* race 2 strain BS3127 to 364 for the *Xhv* race 1 strain BP5179, with an average of 180 contigs per strain. The N50 for *Xhv* strains varied from 27,000 to 129,000 bp. Whole genome annotation showed that each *Xhv* strain contained about 4,700 coding sequences (CDS), with an average total length of CDS per genome being 4.7 Mb and accounting for 91% of the total genome size. The number of plasmids predicted for each *Xhv* strain varied from zero to four, with no pattern by race observed.

**FIGURE 2:**
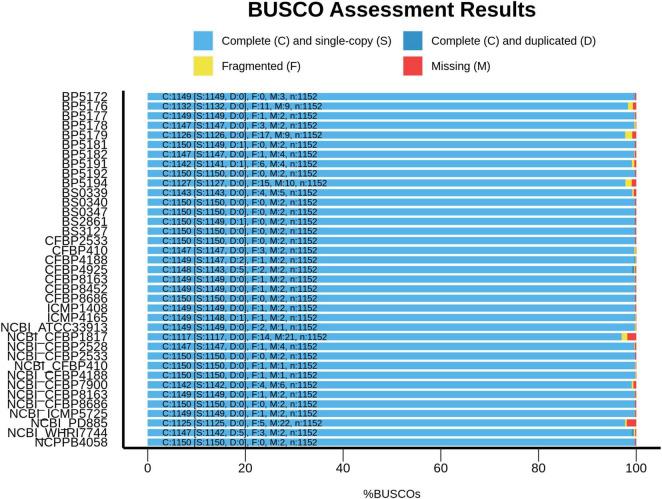
Genome assembly completeness assessment for *Xhv* and related strains. The completeness of the genome assemblies produced in this study was evaluated using the benchmarking universal single copy orthologs method (BUSCO; Manni et al., 2021). The proportions of complete and single-copy orthologs in these assemblies were compared to those of previously published *Xanthomonas* assemblies retrieved from NCBI’s genome database, including *X. campestris* pv. *campestris* (ATCC 33913^T^; GenBank assembly accession GCA_000007145.1), *X. populi* (CFBP 1817^T^; GCA_002940065.1), *X. arboricola* pv. *juglandis* (CFBP 2528^T^; GCA_001013475.1), *X. hortorum* pv. *pelargonii* (CFBP 2533^PT^; GCA_012922215.1), *X. hortorum* pv. *taraxaci* (CFBP 410^PT^; GCA_012922225.1), *X. hortorum* pv. *cynarae* (CFBP 4188^PT^; GCA_002939985.1), *X. hortorum* pv. *carotae* (CFBP 7900; GCA_000505565.1), *X. hortorum* pv. *gardneri* (CFBP 8163^PT^; GCA_012922265.1), *X. hortorum* pv. *vitians* (CFBP 8686^PT^; GCA_012922135.1), *X. campestris* pv. *coriandri* (ICMP 5725^PT^; GCA_019201305.1), *X. fragariae* (PD 885^T^; GCA_900183975.1), and *X. hortorum* pv. *hederae* (WHRI 7744^T^; GCA_003064105.1).

Compared to the *Xhv* strains, the related *Xanthomonas* strains had approximately the same genome sizes, fewer gaps in the sequence with an average of 170 contigs per strain, and similar variation in N50. Annotations for these reference strains revealed similar statistics for coding sequences, with 4,650 CDSs at an average length of 4.7 Mb and representing 91% of the total genome size. A single plasmid is predicted for each of the reference strains except for the pathotype of *X. campestris* pv. *coriandri*, which did not have any predicted plasmid sequence. BUSCO analysis for measuring the completeness of all of these sequence assemblies showed that they are complete and similar to other published *Xanthomonas* genomes (Figure 2).

### Phylogenetic Analysis

**FIGURE 3:**
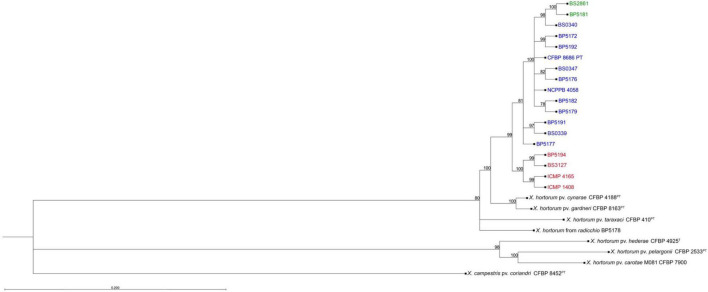
Phylogenetic tree constructed from whole genome SNP data. Maximum-likelihood phylogeny of *Xanthomonas hortorum* pv. *vitians* strains of race 1 (blue), race 2 (red), and race 3 (green) and related *Xanthomonas* type and pathotype strains (black) used in this study. SNP data was extracted from trimmed Illumina sequence reads and core aligned using the snippy-multi pipeline (Seemann, 2015) and the tree was built from sequence alignment using CLC Genomics Workbench. This phylogeny was constructed using the neighbor-joining clustering method and the general time-reversible substitution method with rate variation (4 categories) and estimated topology. Bootstrap values are shown above branches and branch length represents the expected number of nucleotide substitutions per site. Branches shorter than 0.0100 are shown as having a length of 0.0100.

The maximum likelihood phylogeny using whole genome SNP data showed a distinct clade to which all the *Xhv* strains belong (Figure 3). Within this larger clade there were three groups that corresponded to the three *Xhv* races, originally distinguished by their differing host reactions on various lettuce plant introduction lines (Bull et al., 2016). *Xhv* race 1 and 2 appear to have diverged from a common ancestor that itself diverged from strains of *X. hortorum* pv. *gardneri* and *X. hortorum* pv. *cynarae*, which are pathogenic on tomato and artichoke, respectively. The *Xhv* race 3 strains appeared to be more recently evolved, stemming from *Xhv* race 1 strains. This corroborated the finding of Fayette et al. (2016) in which *Xhv* race 3 strains made up a single MLST. The relationship between the *Xhv* strains and the other strains of *Xanthomonas hortorum* corroborated results presented in Morinière et al. (2020), which were based on a predicted core proteome rather than the whole genome SNP data presented here.

### Effector Repertoire Variations

The focus of this study was to identify race-specific avirulence genes among the *Xhv* strains that could explain the race-specific HR phenotypes observed upon inoculation into various lettuce-cultivars. Of the total sixty-seven effector homologs predicted, fifteen were identified in all *X. hortorum* strain sequences tested here and constitute the core effector repertoire of the species (Table 3). Regarding the *Xhv* strains, thirty-four of the predicted effector homologs were present in all *Xhv* strains tested, representing the core repertoire of the pathovar. Homologs for the transcription-activator like effector (TALE) *avrHah1* were identified in all *Xhv* strains tested here, as well as in the *X. hortorum* pv. *hederae*, *X. hortorum* pv. *taraxaci*, *X. hortorum* pv. *gardneri*, *X. hortorum* pv. *cynarae*, *and X. campestris* pv. *coriandri* sequences evaluated. This class of effectors is significant for its ability to enter the host cell nucleus and alter transcription in such a way that makes the host susceptible to disease (Bogdanove et al., 2010). Although race-specific variation in *avrHah1* sequence was not observed in our strains, this could be a limitation in the ability of our short-read sequencing method to resolve the repeat sequences characteristic of TALEs. Further research is needed using a long-read sequencing platform that can demonstrate whether there are race-specific differences as well as pathovar-specific differences in the *avrHah1* gene.

**TABLE 3:**
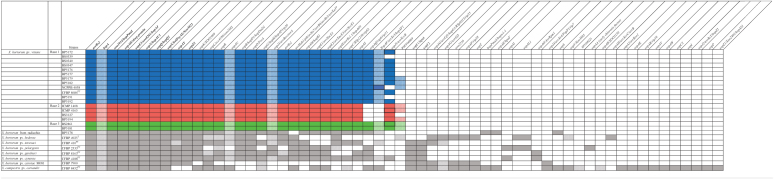
Filtered matches for effector sequences in *Xhv* and related *Xanthomonas s*trains.

*Bolded effectors are those predicted for all X. hortorum strains. Those effectors with an asterisk indicate that they are TALEs. Solid color indicates a match of >60% identity, >40% qcovhsp, and >0.00001 e-value; striped color lowers the percent identity filter to >45%; and no fill corresponds to absence of that effector gene. Blue color corresponds to Xhv race 1 hits, red to Xhv race 2 hits, green to Xhv race 3 hits, and gray to related Xanthomonas reference strain hits. Thick box borders indicate gene presence verified through targeted gene amplification and visualization by gel electrophoresis.*

Two additional effectors, *xopAF* and *xopAQ*, were predicted in the genomes of all *Xhv* strains belonging to races 1 and 3 but were absent from those of *Xhv* race 2 strains. The presence of *xopAF* was also predicted in the genomes of *X. hortorum* pv. *taraxaci*, *X. hortorum* pv. *cynarae*, and *X. campestris* pv. *coriandri* but not in the genomes of *X. hortorum* pv. *pelargonii*, *X. hortorum* pv. *gardneri*, *X. hortorum* pv. *carotae* M081, or the *X. hortorum* strain from radicchio. Further, the presence of *xopAQ* was predicted in the genomes of *X. hortorum* from radicchio, *X. hortorum* pv. *pelargonii*, *X. hortorum* pv. *cynarae*, and *X. campestris* pv. *coriandri* but not of *X. hortorum* pv. *hederae*, *X. hortorum* pv. *taraxaci*, or *X. hortorum* pv. *carotae* M081.

The gene *xopAF* was first identified as *avrXv3* in *X. euvesicatoria* pv. *perforans* strains of tomato race 3 (T3) and was demonstrated to be responsible for race-specific HR elicitation in tomato cultivar Hawaii 7981 (Astua-Monge et al., 2000). Acting intracellularly, *xopAF* causes the upregulation of defense related genes (Balaji et al., 2007). Since its original identification, homologs have been identified in strains of *X. citri* pv. *citri* (*Xcc*) isolated only from Mexican lime and *X. vasicola* pv. *vasculorum* (*Xvv*) isolated only from sugarcane. Experiments with *xopAF* mutants showed that the gene was not responsible for limiting the host range of *Xcc*, but did contribute to virulence (Jalan et al., 2013). Cultivar-specificity for *Xvv* strains does not appear to have been tested.

The other gene, *xopAQ*, was first identified as *rip6/rip11* in a screen for *Ralstonia solanacearum* proteins that are injected into host plant cells (Mukaihara et al., 2010). The *Xanthomonas* homolog *xopAQ* was first described in *X. hortorum* pv. *gardneri* and hypothesized to be part of a group of pathotype-specific genes responsible for its aggressive disease compatibility on tomato and pepper (Potnis et al., 2011). Homologs have since been found in *X. arboricola*, *X. citri*, and *X. euvesicatoria* and have a proposed function in lipid modification that may be involved in host recognition and hypersensitive response (Thieme et al., 2007; Barak et al., 2016).

Five of the effectors predicted for all *Xhv* strains were found within their plasmid sequences: *avrBs1*, *avrHah1*, *xopE1*, *xopE2*, and *xopH* (Supplementary Data 6). Several other effector genes were predicted among various *Xhv* strains, including *avrBs2*, *xopL*, *xopJ5*, *xopAQ*, *xopB*, *xopC*, *xopD*, *xopG*, and *xopZ2*, but with no pattern that could underly the race-specific differences in HR elicitation in lettuce cultivars. Each of the reference strains had a different predicted repertoire of plasmid-borne effectors, and there were no plasmid-borne effectors that were predicted for all *X. hortorum* pathovars. The genes *avrBs1*, *avrHah1*, *xopH*, *xopAQ, avrBsT* (predicted only in *X. campestris* pv. *coriandri*), and *xopAO* (predicted only in *X. hortorum* pv. *gardneri* and *X. hortorum* pv. *cynarae*) are known to be plasmid-borne in other *Xanthomonas* species (Swanson et al., 1988; Minsavage et al., 1990; Schornack et al., 2008; Potnis et al., 2011; Newberry et al., 2019; Roach et al., 2019), and so were likely acquired through plasmid transfer in the strains tested here. All other predicted plasmid-borne effectors (see Supplementary Data 6 for the complete list) are not yet demonstrated to be plasmid-borne in other species but may still have been acquired through plasmid transfer to a strain ancestral to *X. hortorum* pv. *vitians*. Further study with completed *Xhv* genomes produced using long-read sequencing may be able to address these questions regarding the acquisition of these effectors. All effectors not predicted on plasmid sequence here are assumed to be chromosomal genes.

From these data we hypothesize that the differences in effector repertoire composition between *Xhv* races 1 and 3, and *Xhv* race 2, is responsible for the race-specific elicitation of HR in lettuce. It is possible that *xopAQ* and/or *xopAF* interact with an R-gene in ARM-09-161-10-1 to trigger resistance to *Xhv* race 1 and 3 strains, and that the absence of these genes in *Xhv* race 2 strains allows them to go undetected. Further work is needed to confirm the avirulence function of these *xopAQ* and *xopAF* homologs in ARM-09-161-10-1, and that the resistance in these cultivars is governed by a single dominant R gene.

### XopAQ Homolog and New XopAF2 Variant

**FIGURE 4:**
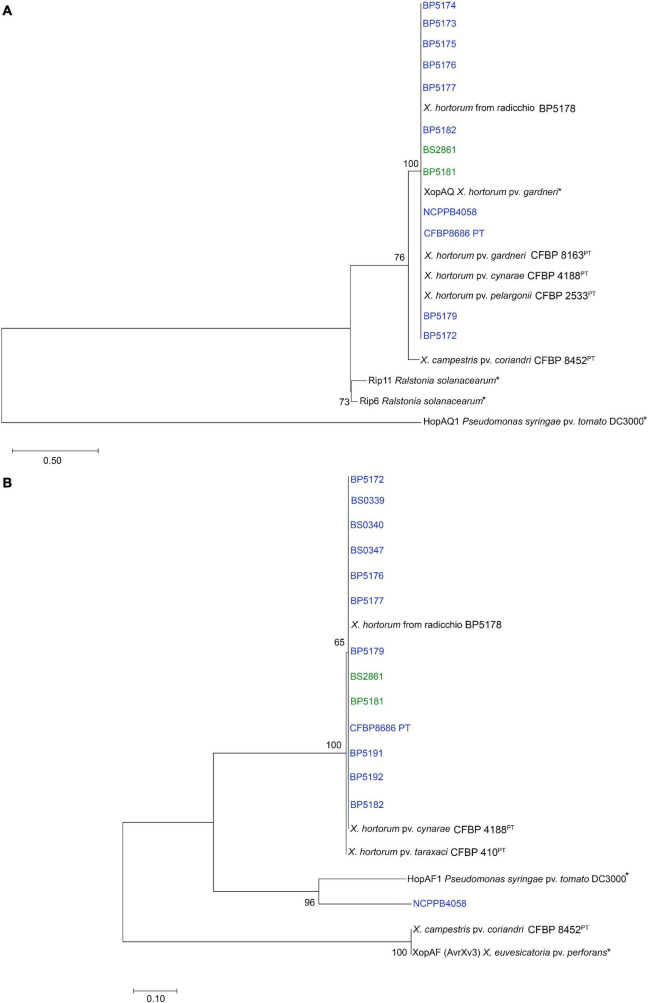
Phylogenetic trees constructed from (A) XopAQ amino acid alignment and (B) XopAF amino acid alignment. Maximum-likelihood phylogenies were constructed for the two genes present in *Xhv* race 1 and race 3 strains (blue and green) but missing from *Xhv* race 2 strains. Nucleotide sequences for these genes were extracted from the whole genome sequence assemblies and converted to amino acid sequences using OrthoFinder. Alignments and trimming were completed using MEGA11, as well as the phylogeny building using the Jones-Taylor-Thornton (JTT) substitution model, uniform substitution rates among sites, and nearest-neighbor-interchange (NNI) method for tree inference. Bootstrap values are shown beside branches and branch length represents the expected number of amino acid substitutions per site. Asterisks indicate published sequences used as references for proteins HopAF1 and XopAF (Astua-Monge et al., 2000; Petnicki-Ocwieja et al., 2002) and XopAQ, HopAQ1, Rip6, and Rip11 (Guttman et al., 2002; Mukaihara et al., 2010; Potnis et al., 2011).

Alignment of the XopAQ protein sequences revealed that they were present among X. *hortorum* strains (Table 3), it was identical to XopAQ protein sequence from *X. hortorum* pv. *gardneri*. In the gene tree, all XopAQ protein sequences from *X. hortorum* strains clustered together and were separated from the HopAQ1 protein sequences from *Pst* DC3000 (Figure 4A).

The XopAF protein sequence alignment showed that all *Xhv* race 1 and 3 homologs were identical except for that of NCPPB 4058, which had significant variation. Phylogenetic analysis revealed that all XopAF protein sequences from *X. hortorum* strains clustered together and were more closely related to the HopAF1 protein sequences from *Pseudomonas syringae* pv. *tomato* (*Pst*) DC3000 than the XopAF/AvrXv3 from *Xanthomonas euvesicatoria* pv. *perforans* (Figure 4B). It is possible this effector gene was originally acquired by the ancestor of *X. hortorum* complex from a *Pseudomonas* species. Here, we designate this new effector variant XopAF2 for *X. hortorum* and propose to distinguish it from protein XopAF1 identified in *X. euvesicatoria*.

Determining the regions flanking *xopAQ* and *xopAF2* in *Xhv* race 1 and 3 strains allowed for the identification of those same regions in *Xhv* race 2 strains, and alignments revealed the gene sequence present in place of those effector genes. Where *xopAQ* would have been present, the *Xhv* race 2 strains vary in the sequence at this position: conserved domain search revealed that ICMP 1408 encoded for a DNA breaking-rejoining enzyme, while ICMP 4165 encoded for an IS3 family transposase and BS3127 did not encode any conserved domain. In contrast, where *xopAF* would have been present, alignments showed that the *Xhv* race 2 strains all had the same sequence which was predicted to encode a glycosyl hydrolase family 3 C-terminal domain. Mining the *Xhv* race 1 and 3 genomes showed that this sequence could be found elsewhere in those genomes, and so its presence alone is not likely a distinguishing feature of a particular *Xhv* race.

In comparison to *Xhv* race 2 strain BS3127, the number of insertion sequences present in *Xhv* race 1 and 3 strains ranged from 64 to 111, with an average of 92 (Table 2). Searching these insertion sequences for effector genes yielded no matches using the same effector gene mining method used for the whole genome sequences. Searches for the transposase genes included in the ISEScan package reveals a negative sense transposase sequence on the same node as *xopAF* and 953 bp downstream. This transposase sequence belongs to insertion sequence family IS5, and the sequence itself is 371 bp in length. Further, searching the *Xhv* race 1 and 3 insertion sequences for phage elements using PHASTER reveals two intact prophage regions. These results indicate that the effector gene *xopAF* may have been acquired by a strain ancestral to *Xhv* race 1 and 3 strains through phage-mediated gene transfer. As expected, no insertion sequences were found in other *Xhv* race 2 strains when compared to BS3127, which supports the assertion that these strains are more related to one another than to the *Xhv* race 1 and 3 strains.

### Secondary Metabolites

Though the main interest in this study was to identify effector repertoires as possible genetic sources of host-specificity, several other virulence factors were investigated for genetic differences among the strains tested that could be useful for characterization of the *Xhv* races. The secondary metabolites Xanthomonadin and Xanthoferrin are encoded by gene clusters that are conserved among *Xanthomonas* spp. Xanthomonadin was first described in *X. oryzae* pv. *oryzae* (*Xoo*) and is a yellow pigment that protects bacteria from the increase in lipid-degrading free radicals caused by UV exposure (Rajagopal et al., 1997; Ayala et al., 2014). Xanthoferrin is a siderophore that enhances bacterial survival in low-iron conditions, such as the plant epidermis, due to its iron search and uptake function. Knock-out mutants for these metabolites resulted in decreased virulence for *X. campestris* pv. *campestris*, the causal agent of black rot on cabbage. For Xanthomonadin, lipid peroxidation is higher in knock-out mutants than in WT strains (Rajagopal et al., 1997). For Xanthoferrin, the knock-out results in smaller leaf lesions and less migratory capacity through the tissue (Pandey et al., 2017).

Secondary metabolite gene cluster mining of the whole genome sequences using antiSMASH revealed the possible presence of two clusters (Table 4). Nine to eleven genes of the 14-gene cluster encoding Xanthomonadin I, as described for *Xathomonas oryzae* pv. *oryzae* (*Xoo*), were found only in the genome sequences of *Xhv* race 1 strains BP5172 and BS0340, and those of the reference strains of *X. hortorum* from radicchio and *X. hortorum* pv. *hederae*. If functional, the production of Xanthomonadin I would afford these strains a fitness advantage in their ability to resist UV damage. The entire 7-gene cluster for Xanthoferrin production, as described for *Xoo*, was found in all genome sequences tested except for those of *Xhv* race 1 strain BP5179, the *X. hortorum* pv. *hederae* pathotype, and the *X. hortorum* pv. *taraxaci* pathotype, which were missing one gene, *XOO1360*. The strains that do produce Xanthoferrin would be better equipped to survive in low-iron conditions and may be able to spread further through host tissues than strains that do not produce Xanthoferrin. Further research is necessary to demonstrate the production and activity of these metabolites empirically.

**TABLE 4:** Predicted secondary metabolite gene clusters.

	Type	Aryl polyene	Lasso peptide	Siderophore

	Strains	Xanthomonadin I	Xanthomonin I/II	Xanthoferrin
*X. hortorum* pv. *vitians* race 1	BP5172	11/14	4/4	7/7
	BS0339	0/14	4/4	7/7
	BS0340	10/14	4/4	7/7
	BS0347	0/14	4/4	7/7
	BP5176	0/14	4/4	7/7 (split contigs)
	BP5177	0/14	4/4	7/7
	BP5179	0/14	4/4	6/7
	BP5182	0/14	4/4	7/7
	NCPPB 4058	0/14	4/4	7/7
	CFBP 8686^PT^	0/14	4/4	7/7
	BP5191	0/14	4/4	7/7
	BP5192	0/14	4/4	7/7
*X. hortorum* pv. *vitians* race 2	ICMP 1408	0/14	4/4	7/7
	ICMP 4165	0/14	4/4	7/7
	BS3127	0/14	4/4	7/7
	BP5194	0/14	4/4	7/7
*X. hortorum* pv. *vitians* race 3	BS2861	0/14	4/4	7/7
	BP5181	0/14	4/4	7/7
*X. hortorum* from radicchio	BP5178	10/14	4/4	7/7
*X. hortorum* pv. *hederae*	CFBP 4925^T^	10/14	2/4	6/7
*X. hortorum* pv. *taraxaci*	CFBP 410^PT^	9/14	4/4	6/7
*X. hortorum* pv. *pelargonii*	CFBP 2533^PT^	10/14	4/4	7/7
*X. hortorum* pv. *gardneri*	CFBP 8163^PT^	11/14	4/4	7/7
*X. hortorum* pv. *cynarae*	CFBP 4188^PT^	0/14	4/4	7/7
*X. hortorum* pv. *carotae*	CFBP 7900	0/14	4/4	7/7
*X. campestris* pv. *coriandri*	CFBP 8452^PT^	10/14	0/14	7/7

*Solid color indicates predicted gene presence, no fill indicates predicted gene absence. Blue color corresponds to Xhv race 1 hits, red to Xhv race 2 hits, green to Xhv race 3 hits, and gray to related Xanthomonas reference strain hits. Fractions indicate the observed number of genes over the expected number of genes for that cluster.*

### Bacteriocins

Bacteriocins are also potential virulence factors. Three classes of bacteriocins have been proposed: class I which are heat-stable and ribosomally produced, post-translationally modified peptides (RiPPs); class II which are heat-stable and unmodified; and class III which are thermolabile and unmodified. Xanthomonin I/II, microcin, and rhodanodin are all class I bacteriocins called lasso peptides, so named for their 3-D looped structure (Hegemann et al., 2013). Sactipeptides and lanthipeptides also belong to class I, and all class I peptides have a variety of functions and may aid in bacterial fitness *via* antibacterial or antiviral activity (Maksimov et al., 2012). Zoocin A is a class III bacteriocin known as a bacteriolysin, which also serves as an antimicrobial peptide but differs from the others in its ability to degrade target bacterial cell walls (Simmonds et al., 1996).

Searches for bacteriocin gene clusters using antiSMASH revealed the presence of one bacteriocin among the strains tested (Table 4), and a parallel analysis using BAGEL4 produced more detailed as well as some contradictory results (Table 5). The entire 4-gene cluster encoding Xanthomonin I/II, as previously described for *Xanthomonas hortorum* pv. *gardneri* (ATCC 19865), was found in all strains except the *X. hortorum* pv. *hederae* pathotype which was missing half of the cluster, and *Xanthomonas campestris* pv. *coriandri*, which was missing the entire cluster. This might suggest that all strains except *X. hortorum* pv. *hederae* have an advantage in bacterial fitness due to their production of Xanthomonin I/II. However, searches with BAGEL4 revealed that five of the *Xhv* race 1, two of the *Xhv* race 2, and both *Xhv* race 3 strains encoded for the Xanthomonin I cluster, and the remaining seven *Xhv* race 1 and two of the *Xhv* race 2 strains encoded for the Xanthomonin II cluster. Among the non-*Xhv* strains, *X. hortorum* from radicchio, *X. hortorum* pv. *hederae*, *X. hortorum* pv. *taraxaci*, *X. hortorum* pv. *pelargonii*, and *X. hortorum* pv. *cynarae* all encoded for the Xanthomonin I cluster; *X. hortorum* pv. *gardneri* and *X. hortorum* pv. *carotae* M081 encoded for the Xanthomonin II cluster. *X. campestris* pv. *coriandri* did not encode for either Xanthomonin cluster. These results suggest that all strains tested except *X. campestris* pv. *coriandri* might lack the fitness advantage afforded by Xanthomonin I/II production, however further study would be necessary to determine whether Xanthomonin I or II is expressed by these strains and confer a fitness advantage.

**TABLE 5:** Predicted bacteriocin genes.

	Class:	I	I	I	I	I	I	II	III

		Lasso peptide	Lasso peptide	Lasso peptide	Lasso peptide	Sactipeptides	Lanthipeptide	Leaderless	Bacteriolysin

		Xanthomonin I	Xanthomonin II	Microcin	Rhodandoin	Sactipeptides	Lanthipeptide	Enterocin	Zoocin A
	**Strains**								
*X. hortorum* pv. *vitians* race 1	BP5172								
	BS0339								
	BS0340								
	BS0347								
	BP5176								
	BP5177								
	BP5179								
	BP5182								
	NCPPB 4058								
	CFBP 8686^PT^								
	BP5191								
	BP5192								
X. *hortorum* pv. *vitians* race 2	ICMP 1408								
	ICMP 4165								
	BS3127								
	BP5194								
X. *hortorum* pv. *vitians* race 3	BS2861								
	BP5181								
*X. hortorum* from radicchio	BP5178								
*X. hortorum* pv. *hederae*	CFBP 4925^T^								
*X. hortorum* pv. *taraxaci*	CFBP 410^PT^								
*X. hortorum* pv. *pelargonii*	CFBP 2533^PT^								
*X. hortorum* pv. *gardneri*	CFBP 8163^PT^								
*X. hortorum* pv. *cynarae*	CFBP 4188^PT^								
*X. hortorum* pv. *carotae*	CFBP 7900								
*X. campestris* pv. *coriandri*	CFBP 8452^PT^								

*Solid color indicates predicted gene presence, no fill indicates predicted gene absence. Blue color corresponds to Xhv race 1 hits, red to Xhv race 2 hits, green to Xhv race 3 hits, and gray to related Xanthomonas reference strain hits.*

Several other bacteriocins were found to be encoded in the strains tested using BAGEL4. A cluster encoding for microcin production was found in 5 of the 12 *Xhv* race 1 strains, all the *Xhv* race 2 and 3 strains, and all the related *Xanthomonas* strains except for *X. hortorum* pv. *hederae* and *X. hortorum* pv. *taraxaci*. *X. hortorum* pv. *hederae*^PT^ and *X. hortorum* pv. *carotae* M081 were the only strains encoding for rhodandoin and lanthipeptide, respectively. Nine *Xhv* race 1 strains, all the *Xhv* race 2 and 3 strains, *X. hortorum* from radicchio, *X. hortorum* pv. *hederae*, and *X. hortorum* pv. *taraxaci* encoded for sactipeptide. For each strain in which they are produced, these class I bacteriocins likely enhance that strain’s ability to compete for resources through antibacterial and antiviral activity. All strains tested encoded for zoocin A production, a type of class III bacteriocin known as a bacteriolysin. The presence of this cluster in all strains suggests that they have a fitness advantage due to the ability to degrade the cell walls of competitor bacteria.

### Type II Secretion System

The type II, type III, type IV, and type VI bacterial secretion systems are important delivery systems for pathogenicity and virulence factors, and so the whole genome sequence data was also used to assess the possible presence and composition of these secretions system gene clusters. Type II secretion systems (T2SSs) enhance the virulence of plant pathogens by releasing cell wall degrading enzymes and toxins (Jha et al., 2005). The secretion complex is composed of an inner membrane platform, a pseudopilin extending from that platform into the periplasm, and a secretin forming a pore in the outer membrane. Its substrates are transported into the periplasm by the Sec or Tat pathways and into the path of the type II pseudopilin, which then pushes the substrate out through the outer membrane pore. There are two variations of the type II secretion system, known as Xps and Xcs; although only the former contributes to virulence, the latter has some homology that allows for it to complement the former. Both variations can be present in the same strain, or the Xps system may be present alone, and they are both encoded in gene clusters (*xpsE-N*, *xpsD*; *xcsC-N*, respectively).

**FIGURE 5:**
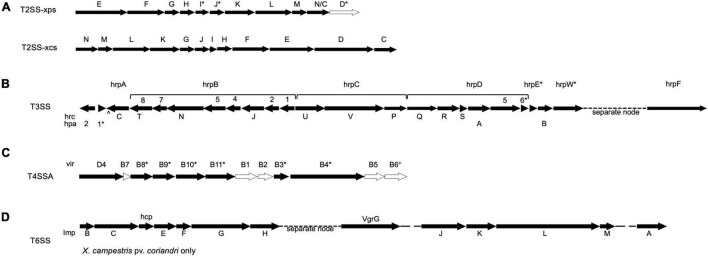
Predicted Secretion system gene clusters for *Xhv* and related *Xanthomonas* strains. Presence of each labeled gene in the majority of *Xhv* and related strains is indicated by a black arrow, and absence from all strains is indicated by a white arrow. Asterisks, carets, and crosses indicate exceptions listed here. **(A)** T2SSs xps and xcs—exceptions include *X. hortorum* pv. *taraxaci* and *X. hortorum* pv. *gardneri*, for which the entire xps gene cluster is predicted, and *Xhv* race 1 strain BP5179, for which the genes *xpsI*, *xpsJ*, and *xpsD* were truncated; **(B)** T3SS—exceptions include *X. hortorum* pv. *hederae*, which had a large insertion between *hpa1* and *hrcC* and lacked *hrpE*, *X. campestris* pv. *coriandri*, which lacked *hpa1* and *hrpW*, and *X. hortorum* pv. *pelargonii*, which lacked *hrpE* and *hrpD6*; **(C)** T4SSA—exceptions include *Xhv* race 1 strains BS0541 and BS3044, which encoded the additional gene *virB6*, *X. hortorum* pv. *gardneri*, which encoded the additional *virB6* gene and lacked *virB11*, *X. hortorum* from radicchio, which only encoded for *virD4*, and *X. campestris* pv. *coriandri*, which only encoded for *virB4*, *virB8*, *virB9*, and *virD4*; and **(D)** T6SS—which was only predicted for the *X. campestris* pv. *coriandri* strain and not found in any other strain tested.

T2SS gene cluster mining of each of the 25 whole genome sequences included in this study revealed that all encoded for the complete Xcs-T2SS (Figure 5A). However, the *xcs* gene cluster has not been implicated in the virulence of a plant pathogen to date, except in its ability to complement the *xpsD-H* genes of the Xps system, and so it is unlikely to play a role in virulence for the strains in included in this study. Regarding the *xps* gene cluster, *X. hortorum* pv. *taraxaci* and *X. hortorum* pv. *gardneri* genomes encoded for its entirety, and all the other genomes encoded for the entire cluster minus the *xpsD* gene (Figure 5A). One exception was the *Xhv* race 1 strain BP5179 genome sequence, which lacked *xpsI* and parts of *xpsJ* and *xpsD*—all of which may be explained by gaps between nodes at their expected positions in the sequence. The incomplete or absent *xpsD* genes may be complemented by the presence of *xcsD* due to significant homology. No T2SS genes were identified within predicted plasmid sequences, suggesting that these genes are chromosomal. Overall, these results suggest that all strains studied here contained a functional Xps-T2SS that may contribute to virulence through the delivery of toxins and cell-wall degrading enzymes, though empirical study is necessary to confirm this hypothesis.

### Type III Secretion System

Type III secretion systems (T3SSs) play a major role in the virulence and pathogenicity of bacterial plant pathogens (Büttner, 2016). The transmembrane complex includes a needle-like structure that extends into the extracellular space and can pierce the host plant cell wall to directly deliver effector proteins into the host cell cytoplasm. Once inside the host cell, the effectors can halt the basal immune response, tag R-proteins for degradation, disrupt protective phytohormones, or enter the nucleus and alter gene transcription—all of these are strategies to enhance virulence or enable pathogenicity. A cluster of approximately 20 genes encodes the T3SS (Rossier et al., 1999). The effector genes that they translocate are usually dispersed throughout the genome and near to mobile genetic elements, which facilitates their transfer and potential for gain or loss (Darrasse et al., 2013; Cesbron et al., 2015).

Twenty-four genes contributing to the T3SS structure and function were queried against the 25 *Xanthomonas* strains included in this study, and matches are demonstrated in Figure 5B. The genome sequences of the *X. hortorum* pv. *vitians* strains, *X. hortorum* from radicchio, *X. hortorum* pv. *taraxaci*, *X. hortorum* pv. *gardneri*, and *X. hortorum* pv. *cynarae* all encoded for the complete hrp gene cluster and likely have functional systems (Hueck, 1998). The *X. campestris* pv. *coriandri* genome sequence tested here lacked *hpa1* and *hrpW.* The *hpa1* gene product transports effectors across the T3SS apparatus in *X. oryzae* pv. *oryzae* (Wang et al., 2018) and the *hrpW* gene is a type III secreted protein that resembles both a harpin and pectate lyase (Charkowski et al., 1998). Either of these two genes may contribute to virulence, but they are not required for a functional T3SS; therefore, this *X. campestris* pv. *coriandri* strain likely has a functional T3SS. On the other hand, the genome sequences of *X. hortorum* pv. *hederae* and *X. hortorum* pv. *pelargonii* both lacked *hrpE*, and that of *X. hortorum* pv. *pelargonii* also lacked *hrpD6*. The *hrpE* gene encoded the type 3 pilus in *X. vesicatoria* (Weber et al., 2005) and the *hrpD6* gene was responsible for regulating the expression of other T3SS structural genes and type three secreted effectors in *X. oryzae* pv. *oryzicola* (Li et al., 2011); without these genes the T3SS is likely not functional in these strains. Further research would be necessary to demonstrate the translocation activity of these secretion systems empirically, especially for *X. hortorum* pv. *hederae* and *X. hortorum* pv. *pelargonii*, which both encode T3Es but seem to lack key T3SS structural gene sequences in their genomes. No T3SS genes were found to be encoded by predicted plasmid sequences, indicating that they are chromosomal.

### Type IV Secretion System

There are two classes of type IV secretion systems (T4SSs); class A has 12 core genes (*virB1-11* and *virD4*; Tzfira and Citovsky, 2006) and class B has 5 core genes (*Dot*/*IcmCDFG*[*H*/*K*]) and up to 27 total genes (Hilbi et al., 2017; Qiu and Luo, 2017). Class A substrates have antimicrobial activity to reduce competition for resources; Class B substrates interfere with plant host cell signaling. Class B has not yet been found in *Xanthomonas* spp. The database used here for type IV secretion system gene mining only included the 10 class A core genes for which DNA sequences are available.

All genome sequences tested here encoded for incomplete T4SSs of class A (Figure 5C). The *Xhv* genome sequences and those of *X. hortorum* pv. *hederae*, *X. hortorum* pv. *pelargonii*, and *X. hortorum* pv. *taraxaci*, all encoded for the genes *virB3*, *virB4*, *virB8*, *virB9*, *virB10*, *virB11*, and *virD4*, and those of two *Xhv* race 1 strains also encoded for *virB6* (BP5177 and NCPPB 4058). For three of the *Xhv* race 2 strains (ICMP 4165, BS3127, and BP5194), *virD4* was encoded for on predicted plasmid sequence and all other predicted T4SS genes were predicted on chromosomal sequence (Supplementary Data 6). In other *Xanthomonas* species, T4SS the genes *virB1*, *virB2*, *virB4*, *virB9*, *virB10*, *virD2*, and *virD4* have been shown to be plasmid-borne, and among those all but *virD2* can be found duplicated in a larger T4SS gene cluster located on the chromosome (Potnis et al., 2011). The genomic sequence of *X. hortorum* pv. *gardneri* encoded for nearly the same T4SS composition as the *Xhv* strains but was missing *virB11* and had the additional *virB6* gene. The genome sequence of *X. hortorum* isolated from radicchio encoded for only *virD4*. *X. campestris* pv. *coriandri* genome sequence encoded for only *virB4, virB8*, *virB9*, and *virD4*. All chromosomal and plasmid sequences assessed here lacked genes that encoded the core complex and pilus of this system (Sgro et al., 2019). Without core structural genes, it is likely that none of these strains have functional T4SSs. More research is necessary to confirm this hypothesis.

### Type VI Secretion System

The type VI secretion system (T6SS) in plant pathogenic bacteria has antimicrobial ability; they are used to directly inject toxins into competitor bacteria cells to reduce competition for resources (Bernal et al., 2018). The contribution of T6SS in plant host interactions, however, has not yet been investigated (Liyanapathiranage et al., 2021). A cluster of thirteen type six secretion genes, *tssA-tssM*, encode the system and were formerly referred to as imp (impaired in nitrogen fixation) genes. The injection structure they form resembles a bacteriophage and toxin delivery relies on its direct contact with bacterial cell walls. The genes *tssD* and *tssI*, also known as *hcp* and *vgrG*, respectively, are thought to be both structural components and substrates of this system (Russell et al., 2014).

The only whole genome sequence included in this study that encoded for a T6SS was *X. campestris* pv. *coriandri* (Figure 5D). The gene cluster was split between two nodes; node 6 contained genes *tssB*, *tssC*, *tssD*, *tssE-H* and node 15 contained genes *tssI-M* and *tssA*. None of these genes were found among predicted plasmid sequences, suggesting that they are all encoded chromosomally. The presence of all core genes suggests that the T6SS is functional in this strain of *X. campestris* pv. *coriandri* and may contribute to its fitness against competitor bacteria.

Secretion system mining revealed that the *X. hortorum* strains, including all *Xhv* strains tested here, likely have functional type II and III secretion systems and do not have type IV or type VI secretion systems. *X. campestris* pv. *coriandri* also likely has functional type II, III, and VI secretion systems but likely lacks the type IV secretion system. The presence of type II and type III secretion system gene clusters suggests that these strains can deliver type II secreted toxins and enzymes and type III secreted effectors that may enhance virulence. In *X. campestris* pv. *coriandri*, the type VI secretion system may offer a fitness advantage in the ability to secrete type VI effectors that target competitor bacteria.

## Conclusion

This study revealed insights into the race structure of *X. hortorum* pv. *vitians*. Phylogenetic analysis showed that strains of *Xhv* races 1 and 3 were more closely related to each other than to strains of *Xhv* race 2. HR induction in ARM-09-161-10-1 upon inoculation with *Xhv* race 1 and 3 strains may have been due to a gene-for-gene interaction, in which a race-specific effector is recognized by a resistance protein expressed by this cultivar. Such a race-specific interaction has been demonstrated for tomato cultivars and three races of *X. euvesicatoria* pv. *perforans* (Astua-Monge et al., 2000). The one TALE identified among the genome sequences tested, *avrHah1*, did not show race-specific variation here, but long-read sequencing to resolve the repeat regions may yet reveal such variation. Two other putative effectors identified in this study are possible candidate genes responsible for race-specificity because they were present only in *Xhv* races 1 and 3 genome sequences and not in *Xhv* race 2 genome sequences. These genes are a *xopAQ* homolog and the novel effector variant *xopAF2.* Antisense transposase sequence downstream from *xopAF2* and the prophage sequence identified among nearby *Xhv* race 1 and 3 insertion sequences suggest that an ancestor of *Xhv* race 1 and 3 strains may have acquired *xopAF2* in a phage-mediated gene transfer. Our ongoing research seeks to demonstrate whether these effectors are responsible for HR induction in ARM-09-161-10-1. Additionally, more study is necessary to identify other possible gene products involved in the incompatible interactions of *Xhv* races 1 and 2 with *Xar1*-containing lettuce cultivars, such as Little Gem, and PI491114, respectively. Furthermore, the close relatedness of the *Xhv* race 1 and 3 strains may make it difficult to identify genotypic differences that explain their different disease phenotypes on lettuce cultivar Little Gem, as none were identified in this study.

This study also provided several insights into *Xanthomonas* genetic diversity. The genome statistics for our assemblies are consistent with what is expected for *Xanthomonas* spp. Secondary metabolite and bacteriocin production varied by strain, and no pattern was found that would be useful for characterizing the *Xhv* races. All strains tested here encoded for type II and type III secretion systems, and *X. campestris* pv. *coriandri* also encoded for a type VI secretion system. These systems all appear to be encoded chromosomally, as none of the genes were identified among predicted plasmid sequences.

Understanding the genetic variation among strains of *Xhv* is crucial for developing effective disease management strategies, especially the breeding of lettuce cultivars with durable resistance. Chemical control is most effective when applied prophylactically, but the sporadic nature of the disease can render this approach unnecessary and costly in years that the pathogen does not appear (Bull et al., 2007). A more efficient strategy is the use of resistant cultivars, especially those with multiple genetic sources of resistance against the different races of *Xhv* (Sandoya et al., 2019). Lettuce cultivars resistant to *Xhv* race 1 strains have already been found (Hayes et al., 2014; Wang et al., 2016) and additional germplasm is being sought (Sandoya et al., submitted manuscript). These resistant cultivars encode R-genes *Xar1* and *Xcvr*, which have been mapped to lettuce chromosome two, but the precise sequence has not yet been determined. The presence of these R-genes suggests a possible gene-for-gene interaction between these cultivars and *Xhv* race 1, and the race-specific interactions of *Xhv* race 2 and 3 with lettuce cultivars PI491114 and ARM-09-161-10-1, respectively, may also be due to gene-for-gene interactions with other yet unidentified R-genes. With knowledge of the specific genes interacting with lettuce hosts to induce HR, research could proceed for the precise identification of R-gene interaction partners and subsequent engineering of lettuce cultivars expressing those R-genes.

## Data Availability Statement

The datasets presented in this study can be found in online repositories. The names of the repository/repositories and accession number(s) can be found in the article/Supplementary Material.

## Author Contributions

ER co-authored grants funding research, conducted the analyses, and wrote the manuscript. NP provided bioinformatic and phytobacterial expertise to direct the research. CB co-wrote grants funding research, designed research hypotheses, mentored Ph.D. candidate ER, and edited the manuscript. All authors contributed to the article and approved the submitted version.

## Conflict of Interest

The authors declare that the research was conducted in the absence of any commercial or financial relationships that could be construed as a potential conflict of interest.

## Publisher’s Note

All claims expressed in this article are solely those of the authors and do not necessarily represent those of their affiliated organizations, or those of the publisher, the editors and the reviewers. Any product that may be evaluated in this article, or claim that may be made by its manufacturer, is not guaranteed or endorsed by the publisher.
