# Corrigendum: Comparative genomic analysis of the lettuce bacterial leaf spot pathogen, *Xanthomonas hortorum* pv. *vitians*, to investigate race specificity

**DOI:** 10.3389/fmicb.2022.1044656

**Published:** 2022-11-15

**Authors:** Emma Rosenthal, Neha Potnis, Carolee T. Bull

**Affiliations:** ^1^Department of Plant Pathology and Environmental Microbiology, Pennsylvania State University, University Park, PA, United States; ^2^Department of Entomology and Plant Pathology, Auburn University, Auburn, AL, United States

**Keywords:** bacterial plant pathogens, plant-microbe interactions, comparative genomics, *Xanthomonas*, effectors

In the published article, there was an error. In all instances, we refer the resistance gene of PI358001-1 as *Xcv1*, but the name should be corrected to *Xcvr*.

A correction has been made to the **Introduction** section, paragraph 1.

The sentence previously stated:

“Another identified source of resistance to *Xhv* race 1 strains isolated from Florida lettuce fields was the *Xcv1* R-gene from *L. serriola* PI358001-1 (Wang et al., 2016).”

The corrected sentence appears below:

“Another identified source of resistance to *Xhv* race 1 strains isolated from Florida lettuce fields was the *Xcvr* R-gene from *L. serriola* PI358001-1 (Wang et al., 2016).”

A correction has been made to the **Introduction** section, paragraph 1.

The sentence previously stated:

“Additional races of the pathogen were designated among those *Xhv* strains that did not result in HR in the *Xar1*- or *Xcv1*-enconding cultivars, but instead triggered HR in either *L. serriola* PI491114 (designated race 2) or *L. serriola* ARM-09-161-10-1 (designated race 3).”

The corrected sentence appears below:

“Additional races of the pathogen were designated among those *Xhv* strains that did not result in HR in the *Xar1*- or *Xcvr*-encoding cultivars, but instead triggered HR in either *L. serriola* PI491114 (designated race 2) or *L. serriola* ARM-09-161-10-1 (designated race 3).”

A correction has been made to the **Introduction** section, paragraph 2.

The sentence previously stated:

“The results of this study corroborated previous work on *Xhv* diversity (Sahin et al., 2003; Fayette et al., 2016), and they found that the strains that induce HR upon injection into the *Xar1*- or *Xcv1*-encoding cultivars all belonged to sequetypes B, D, or E.”

The corrected sentence appears below:

“The results of this study corroborated previous work on *Xhv* diversity (Sahin et al., 2003; Fayette et al., 2016), and they found that the strains that induce HR upon injection into the *Xar1*- or *Xcvr*-encoding cultivars all belonged to sequetypes B, D, or E.”

A correction has been made to the **Introduction** section, paragraph 3.

The sentence previously stated:

“The presence of the R-genes *Xar1* and *Xcv1* in lettuce cultivars capable of HR exclusively to *Xhv* race 1 strains (Hayes et al., 2014; Wang et al., 2016) suggested the possibility that these strains might produce an effector that is recognized by those R-genes.”

The corrected sentence appears below:

“The presence of the R-genes *Xar1* and *Xcvr* in lettuce cultivars capable of HR exclusively to *Xhv* race 1 (Hayes et al., 2014; Wang et al., 2016) suggested the possibility that *Xhv* race 1 might produce an effector that is recognized by those R-genes.”

A correction has been made to the **Conclusion** section, paragraph 3.

The sentence previously stated:

“These resistant cultivars encode R-genes *Xar1* and *Xcv1*, which have been mapped to lettuce chromosome two, but the precise sequence has not yet been determined.”

The corrected sentence appears below:

“These resistant cultivars encode R-genes *Xar1* and *Xcvr*, which have been mapped to lettuce chromosome two, but the precise sequence has not yet been determined.”

The authors apologize for this error and state that this does not change the scientific conclusions of the article in any way. The original article has been updated.

## Publisher's note

All claims expressed in this article are solely those of the authors and do not necessarily represent those of their affiliated organizations, or those of the publisher, the editors and the reviewers. Any product that may be evaluated in this article, or claim that may be made by its manufacturer, is not guaranteed or endorsed by the publisher.
