# Supplementary material for: Comparative Genomic Analysis of the Lettuce Bacterial Leaf Spot Pathogen, Xanthomonas hortorum pv. vitians, to Investigate Race Specificity
Source: Front Microbiol. 2022 Apr 18;13:840311. doi: 10.3389/fmicb.2022.840311 (PMC9062649; doi:10.3389/fmicb.2022.840311)
Supplement: Supplementary file 1 [file Data_Sheet_1.zip › Supplemental Data 1-5.DOCX]

Scripts Referenced in Manuscript

**Supplemental 1: trimming.sh**

#this code will run through the quality control step for processing raw sequence data.

#defines where the script will run and where it will print files to

SCRIPTPATH="$( cd "$(dirname "$0")" ; pwd -P )"

#makes the Raw_Data directory to house your raw sequence data

mkdir -p $SCRIPTPATH/Raw_Data

#moves that data into the new directory

mv $SCRIPTPATH/*.fastq $SCRIPTPATH/Raw_Data

#create a directory for the fastqc reports

mkdir -p $SCRIPTPATH/Raw_Data/fastqc_reports

#generate the qc report for the raw sequence data

for file in $SCRIPTPATH/Raw_Data/*.fastq; do fastqc $file; done

#move all fastqc reports to a single directory

mv $SCRIPTPATH/Raw_Data/*.html $SCRIPTPATH/Raw_Data/*.zip $SCRIPTPATH/Raw_Data/fastqc_reports

#trim away the poor quality data and output to new folder

for R1 in $SCRIPTPATH/Raw_Data/*R1_001.fastq

do

R2=${R1//R1_001.fastq/R2_001.fastq}

R1paired=${R1//.fastq/_paired.fq}

R1unpaired=${R1//.fastq/_unpaired.fq}

R2paired=${R2//.fastq/_paired.fq} R2unpaired=${R2//.fastq/_unpaired.fq}

trimmomatic PE $R1 $R2 $R1paired $R1unpaired $R2paired $R2unpaired ILLUMINACLIP:TruSeq3-PE.fa:2:30:10 SLIDINGWINDOW:4:20 MINLEN:50;

done

#make directory for outputs to go into

mkdir -p $SCRIPTPATH/Trimmed_Data

#move trimmed files to Trimmed_Data

mv $SCRIPTPATH/Raw_Data/*paired.fq $SCRIPTPATH/Trimmed_Data

#generate the qc report for the trimmed sequence data

for file in $SCRIPTPATH/Trimmed_Data/*paired.fq; do fastqc $file; done

#create a directory for these fastqc reports

mkdir -p $SCRIPTPATH/Trimmed_Data/fastqc_reports

#move all the fastqc reports over

mv $SCRIPTPATH/Trimmed_Data/*.html $SCRIPTPATH/Trimmed_Data/*.zip $SCRIPTPATH/Trimmed_Data/fastqc_reports

**Supplemental 2: spades.sh**

#this code is to complete a de novo assembly for each strain using spades for paired end reads.

#Input: trimmed paired end reads. Output: single scaffold.fa assembly file.

#this code is the second step of the WGS procedure, starting with quality control and the trimming.sh.

#defines where the script will run and where it will print files to

SCRIPTPATH="$( cd "$(dirname "$0")" ; pwd -P )"

#defines variables for the two input files

FILE_1=$1

FILE_2=$2

#Makes the directory for the output files

mkdir -p $SCRIPTPATH/Assemblies

#runs the assembly using spades and upends the results to a new directory within Assemblies.

spades.py -1 $1 -2 $2 -o $SCRIPTPATH/Assemblies/$(basename ${1%%.fq}) -t 40 -m 64

#Comment out lines 13 and 16 and uncomment the two below one to run plasmid spades instead of denovo spades.

#mkdir -p $SCRIPTPATH/Plasmid

#spades.py --plasmid -1 $1 -2 $2 -o $SCRIPTPATH/Plasmid/$(basename ${1%%.fq}) -t 40 -m 64

**Supplemental 3: spades_loop.txt**

#This is the file that will run the WGS_spades_denovo.sh script in a loop

#Input: all paired end output files of the QCtrimming.sh script; Output: assembly scaffold.fa file

#to run the assembly script, use $ cat WGS_loop.txt | bash

#defines where the script will run and where it will print files to

SCRIPTPATH="$( cd "$(dirname "$0")" ; pwd -P )"

#runs the assembly script in loop for the 25 strains included in this study.

for i in {1..25}; do r1=*_S${i}_L001_R1_001_paired.fq; r2=*_S${i}_L001_R2_001_paired.fq; bash $SCRIPTPATH/denovo_spades.sh $r1 $r2; done

#runs the plasmid assembly script in loop - comment out line 10 and uncomment below to run plasmid assembly in a loop.

#for i in {1..27}; do r1=*_S${i}_L001_R1_001_paired.fq; r2=*_S${i}_L001_R2_001_paired.fq; bash $SCRIPTPATH/plasmid_spades.sh $r1 $r2; done

**Supplemental 4: effectorgene_mining.sh**

#effector mining script: input includes protein fasta file of type and pathotype strain effector genes and scaffolds from whole genome sequencing.

#to run change the pident or qcovhsp, alter line 19 and re-run a new output folder listed in lines 23 and 24.

#ouputs:

#alignments_[filename].txt

#rawtable_[filename].tsv

#tidytable_[filename].tsv

# Stop if any error is found

set -ueox pipefail

#defines where the script will run and where it will print files to

SCRIPTPATH="$( cd "$(dirname "$0")" ; pwd -P )"

for file in $SCRIPTPATH/BS*.fasta

do

makeblastdb -in $file -dbtype nucl -title -parse_seqids -out $(basename ${file//.fasta/"_DB"})

tblastn -db $(basename ${file//.fasta/"_DB"}) -query all_effectors_removedups.faa > alignments_$(basename ${file//.fasta/".txt"})

tblastn -db $(basename ${file//.fasta/"_DB"}) -query all_effectors_removedups.faa -outfmt "6 qseqid sseqid length pident mismatch qstart qend sstart send qcovs qcovhsp evalue bitscore" > rawtable_$(basename ${file//.fasta/".tsv"})

cat rawtable_$(basename ${file//.fasta/".tsv"}) | awk '$4 >= 60 && $11 >= 40 && $12 < 0.00001 {print;}' | sort | uniq > tidytable_$(basename ${file//.fasta/".tsv"})

cat tidytable_$(basename ${file//.fasta/".tsv"}) | cut -f 1 | uniq | sort > effectorlist_$(basename ${file//.fasta/".txt"})

done

mkdir -p $SCRIPTPATH/effectormining03242021_pident60_qcovhsp40

mv rawtable* alignments* tidytable* effectorlist* $SCRIPTPATH/effectormining03242021_pident60_qcovhsp40

**Supplemental 5: SSgene_mining.sh**

#secretion gene mining script: input includes protein fasta file of IMG-JGI-listed secretion system genes and scaffolds from whole genome sequencing project.

#ouputs:

#alignments_[filename].txt

#rawtable_[filename].tsv

#tidytable_[filename].tsv

# Stop if any error is found

set -ueox pipefail

#defines where the script will run and where it will print files to

SCRIPTPATH="$( cd "$(dirname "$0")" ; pwd -P )"

for file in $SCRIPTPATH/BS*.fasta

do

makeblastdb -in $file -dbtype nucl -title -parse_seqids -out $(basename ${file//.fasta/"_DB"})

blastn -db $(basename ${file//.fasta/"_DB"}) -query Potnis_secretion_system_genes.fasta > alignments_$(basename ${file//.fasta/".txt"})

blastn -db $(basename ${file//.fasta/"_DB"}) -query Potnis_secretion_system_genes.fasta -outfmt "6 qseqid sseqid length qlen pident mismatch qstart qend sstart send qcovs qcovhsp evalue bitscore" | sort > rawtable_$(basename ${file//.fasta/".tsv"})

cat rawtable_$(basename ${file//.fasta/".tsv"}) | awk '$5 >= 65 && $12 >= 65 && $13 < 0.00001 {print;}' | sort | uniq > tidytable_$(basename ${file//.fasta/".tsv"})

done

mkdir -p $SCRIPTPATH/secretion_genes2021_08_11-65

mv rawtable* alignments* tidytable* $SCRIPTPATH/secretion_genes2021_08_11-65
